# Complete Genome Sequence of a Deletion Mutant of Vibrio parahaemolyticus from Pacific White Shrimp (Penaeus vannamei)

**DOI:** 10.1128/genomeA.00544-18

**Published:** 2018-06-21

**Authors:** Siddhartha Kanrar, Arun K. Dhar

**Affiliations:** a Aquaculture Pathology Laboratory, School of Animal and Comparative Biomedical Sciences, The University of Arizona, Tucson, Arizona, USA

## Abstract

Vibrio parahaemolyticus carrying the toxin genes *pirA* and *pirB* causes acute hepatopancreatic necrosis disease in shrimp. A genome sequence of V. parahaemolyticus strain R13 was determined that showed deletions of the entire *pirA* gene and the 5ʹ end of the *pirB* gene and does not cause the disease in experimental challenge.

## GENOME ANNOUNCEMENT

Vibrio parahaemolyticus carrying the toxin genes *pirA* and *pirB* causes acute hepatopancreatic necrosis disease (AHPND) in cultured shrimp (1, 2). The binary toxins pirA and pirB are similar to Photorhabdus insect-related (Pir) toxin (3). The disease has spread from Asia to the Americas, and now into Texas in the United States, causing major economic losses throughout its path (4–6).

Recently, a V. parahaemolyticus strain R13 isolated from Penaeus vannamei shrimp in Latin America that tested PCR negative for the *pirA* gene but positive for the *pirB* gene. In an experimental bioassay using specific-pathogen-free Penaeus vannamei shrimp, the R13 strain did not cause any mortality, whereas AHPND-causing V. parahaemolyticus strain A3 (the reference strain that originated in Vietnam) caused 100% mortality (7).

Total genomic DNA was isolated from V. parahaemolyticus strain R13 using a DNeasy blood and tissue kit (Qiagen, Valencia, CA, USA). Library preparation and long-read sequencing were carried out at Arizona Genomics Institute, The University of Arizona, Tucson. A PacBio 20-kb sequencing library was constructed using the SMRTbell template prep kit 1.0 following the manufacturer’s instructions. The final library was processed for sequencing by using PacBio MagBeads kit v2 with the P6/C4 chemistry and following PacBio protocols (Pacific Biosciences, Menlo Park, CA). Sequencing was performed on a PacBio RS II instrument in one single-molecule real-time (SMRT) cell (v3), and the PacBio hierarchical genome assembly process (HGAP) version 3.0 was used for a *de novo* assembly of the sequence reads (8). An average coverage of 172× was obtained. The genome consists of two chromosomes designated Chr 1 (3,477,006 bp) and Chr 2 (1,818,054 bp) and three plasmids designated pVpR13_71Kb (71,596 bp), pVpR13_55Kb (55,412 bp), and pVpR13_19Kb (19,217 bp). The R13 genome was annotated using the RAST version 2.0 pipeline (9). The complete genome consists of 5,441,285 bp, with G+C contents of 45.3% and with 4,928 coding sequences. There are 173 RNAs, of which 37 are tRNAs and 14 are 5S RNAs. The two chromosomes of R13 have an average nucleotide identity of 98.35% with the chromosomes of V. parahaemolyticus reference strain RMID2210633 (10). No acquired antimicrobial resistance gene was detected in any of the plasmids in the R13 strain. However, using the ResFinder 3.0 software program, a beta-lactam resistance signature was found on chromosome 2 (11). The R13 strain carries type I, type II/IV, and type VI secretion systems based on RAST annotation (9). The toxin genes in the R13 strain are located in the pVpR13_71Kb plasmid. Sequence analysis revealed that the promoter region upstream of *pirA,* the entire open reading frame (ORF) of *pirA*, and part of the 5ʹ-end of the *pirB* ORF is deleted in R13. Since the binary toxins are the virulence factor in AHPND-causing V. parahaemolyticus, deletion of the toxin gene(s) contributed to avirulence in the R13 strain. In summary, V. parahaemolyticus R13 is a unique mutant strain that will be very useful in comparative genomic study for determining the minimal toxin gene sequence needed to cause clinical signs of AHPND in shrimp.

### Accession number(s).

The complete genome sequence of strain R13 has been deposited in NCBI/GenBank under the accession numbers CP028342, CP028343, CP028344, CP028345, and CP028346.
